# ERP topographic analyses from concept to articulation in word production studies

**DOI:** 10.3389/fpsyg.2014.00493

**Published:** 2014-05-27

**Authors:** Marina Laganaro

**Affiliations:** Faculty of Psychology and Educational Sciences, University of GenevaGeneva, Switzerland

**Keywords:** ERP, language production, production latency, picture naming, stimulus-aligned, response-aligned, topographic analysis

## Abstract

Most ERP studies using overt speech production tasks have analyzed fixed time-windows of stimulus-aligned ERPs, not exceeding the fastest production latency. These fixed ERP time-windows may cover the whole speech planning process for fast trials or participants, but only part of the planning processes for trials or participants with production latencies exceeding the analyzed period. Two core questions thus emerge when analysing fixed time-windows in overt language production, namely (1) to what extent do ERPs capture “later” encoding processes, especially phonological and phonetic encoding, and (2) how to account for different production latencies across conditions or individuals. Here we review a methodological approach combining waveform and topographic analyses on integrated stimulus- and response-aligned ERPs according to response latencies in each participant and condition. Then we illustrate the approach with a picture naming task. Crucially for the purpose of the methodological illustration, the separate analysis of fixed stimulus- and response-locked ERPs led to a counter-intuitive result (longer lasting periods of stable global electrophysiological activity for the fastest condition). Coherent results with longer lasting periods of topographic stability in the slower condition only appeared when combining stimulus- and response-aligned ERPs in order to cover the actual word planning time-windows. Thus this combined analysis enabled to disentangle the possible interpretations of the neurophysiological processes underlying differences across conditions observed on waveforms and on topographies in the fixed ERP periods.

## INTRODUCTION

When speakers produce a word, they transform an abstract concept into articulated speech sounds in less than 1 s. This transformation involves a series of mental operations which have been modeled thanks to psycholinguistic experimental chronometric investigations (Dell, 1986; Levelt et al., 1999) and more recently with neuroimaging studies allowing high temporal resolution (electroencephalography, EEG, and magnetoencephalography, MEG). The dynamics of the mental processes involved in planning the production of single words has been estimated in a meta-analysis by Indefrey and Levelt (2004, reanalyzed in Indefrey, 2011), in particular regarding word production in picture naming tasks. In such tasks, visual and conceptual processes are estimated to take place from 0 to about 200 ms after picture presentation, followed by lexical-semantic (lexical selection) processes until about 275 ms. The encoding of the phonological form is thought to occur between 275 and 450 ms after picture onset, followed by phonetic encoding and motor execution. This estimate has been made for production latencies of about 600 ms. However, production latencies often exceed 600 ms even among young adults and they are largely beyond 1000 ms with participants from other populations. The question then is how to compare the dynamics of encoding processes corresponding to variable production latencies and how to capture the actual speech planning processes when production latencies exceed the duration of the stimulus-locked epochs analyzed in event-related potentials (ERPs). After analyzing this problem in more detail, we describe an approach aimed at combining stimulus- and response-locked ERPs to cover the actual production latencies and illustrate it with data from an overt picture naming paradigm.

### TIME-WINDOW OF ANALYZED EPOCHS IN ERP STUDIES ON OVERT LANGUAGE PRODUCTION

The first ERP studies on speech production have used metalinguistic paradigms to avoid overt production because of possible artifacts during motor preparation or execution (see Ganushchak et al., 2011 for a review). Alternatively, ERP studies on word production have used implicit, silent or delayed production paradigms, usually during picture naming tasks (Jescheniak et al., 2002; Cornelissen et al., 2003; Schiller et al., 2003; Vihla et al., 2006; Laganaro et al., 2009). In these studies participants either prepare the word (producing it overtly after a delay falling beyond the analyzed period), or they say the word in their mind, therefore also avoiding possible artifacts due to speech articulation. Although these kinds of paradigms address speech production directly (without having recourse to a metalinguistic task), one may wonder whether in delayed or silent production tasks the same planning processes are executed as in overt production. This question has been addressed by Laganaro and Perret (2011) in a study comparing delayed and immediate production in a picture naming task. ERPs differed between the two paradigms from 350 ms after picture presentation, suggesting that only “early” speech planning processes (up to 350 ms) are captured with delayed production tasks.

Overt “immediate” (not delayed) production paradigms are now widely used in ERP investigations. The most current strategy to avoid artifacts due to motor execution consists in analyzing epochs within a time-period not extending beyond the shortest response latency (Maess et al., 2002; Koester and Schiller, 2008). Other authors have applied specific low-pass filters (above 10 Hz, e.g., Ganushchak and Schiller, 2008) to the EEG signal to exclude EMG frequencies, or have used source-separation methods to separate EEG from EMG signals (Riès et al., 2011). Whatever the strategy adopted to avoid analyzing ERPs close to the response, a *fixed time-window* of stimulus- or response-aligned epochs has been analyzed in most studies.

A core question when analyzing fixed ERP time-windows in a domain in which processing times vary largely across trials and participants is which underlying processes are being captured in the ERP signal which is distant from the lock point. For instance, when stimulus-aligned epochs of 500 ms (Maess et al., 2002; Aristei et al., 2011), 600 ms (Costa et al., 2009; Blackford et al., 2012), or 700 ms (Koester and Schiller, 2008) are analyzed, this captures the whole planning period for fast trials and participants (i.e., those with production latencies not exceeding the analyzed period), but the capture of some processes might be truncated in “slower” trials and participants (i.e., those with production latencies exceeding the analyzed period). Some researchers have previously faced and discussed this problem. In an investigation of masked priming effects during overt picture naming, Blackford et al. (2012) reported that phonologically related primes decreased production latencies relative to unrelated primes, but they did not observe any related ERP modulation. The authors suggested that phonological priming may affect ERPs beyond the analyzed 600 ms period, which was imposed by the fastest production latencies. Hence ERP analyses carried out on fixed-time windows not exceeding the fastest production latencies seem unable to track later encoding processes in slower trials and participants. A further question when analysing fixed ERP epochs is how to capture between-subject and between-item variability. In the following section we will present a procedure aimed at extracting ERPs covering the entire and exact time-period from stimulus to response.

### ANALYSING ERPs FROM STIMULUS TO RESPONSE

**FIGURE 1:**
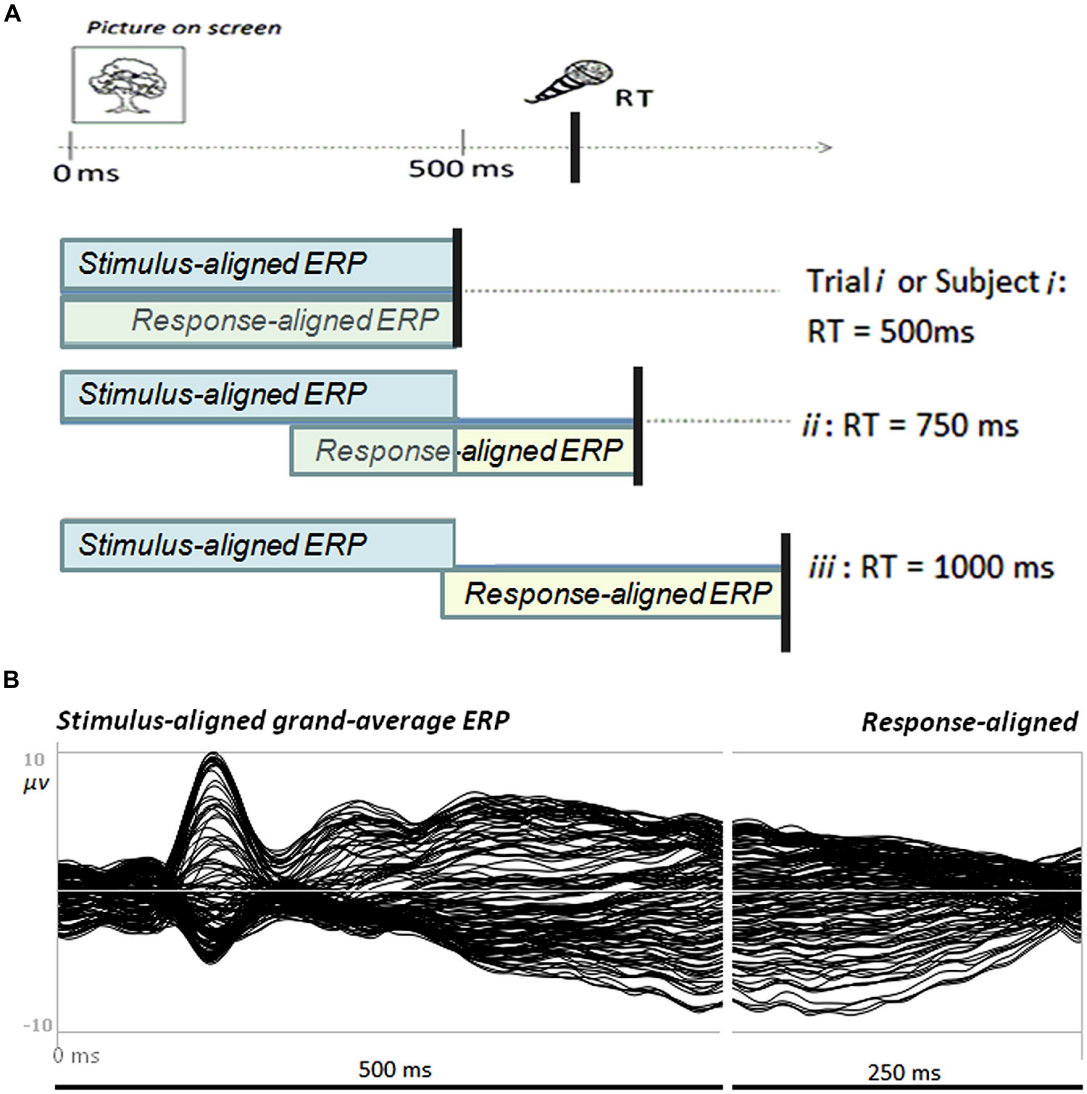
**(A)** Illustration of the time-windows covered by fixed stimulus-locked or response-locked ERP epochs relative to the actual trial or participant production latency (RT) in picture naming task. Only the integration of *stimulus- *and* response-aligned* ERPs covers the period from stimulus onset to articulation onset for all production latencies. **(B)** Example of a grand-average ERP from a picture naming task (butterfly display of 128 electrodes) in which the overlapping signal has been removed from the response-aligned ERP to cover the exact production latency.

It is well known that production latencies can vary in typical picture naming tasks and that the slowest trials or participants may be twice as long as the fastest participants or trials. The problem this variability runs into when analysing fixed ERP time-windows has been described in the previous section and is further illustrated in **Figure 1**. For instance, fixed stimulus-aligned ERPs of 500 ms cover the whole speech planning period for the fastest trials or participants (s1), but not for slower trials or participants (s2, s3). The same holds for backward response-aligned ERPs. One way of obtaining ERP data which cover the whole speech planning period in overt speech production tasks is to combine stimulus- and response-aligned ERPs according to the production latency.

The approach combining stimulus-aligned and response-aligned (backward) ERPs according to each subject’s production latency by removing the overlapping signal has been introduced by Laganaro and Perret (2011) and has been applied to further studies on overt picture naming (Laganaro et al., 2012; Perret and Laganaro, 2012), usually coupled with spatio-temporal (topographic) analyses, as these analyses can be applied to compare ERP signals of variable duration (see details of ERP analyses in the illustration below). Capturing the entire speech planning time-window (from concept to articulation) is of particular value especially when late encoding processes (phonological and phonetic encoding) are investigated or when populations with different production latencies are compared, as for instance healthy controls and brain damaged speakers (Laganaro et al., 2013) or older and younger adults (Valente and Laganaro, submitted). This approach was applied for instance in a study comparing healthy adult participants to brain-damaged patients with impaired phonological-phonetic encoding, the latter group being on average 200 ms slower than the age-matched control participants in the same picture naming task (Laganaro et al., 2013). Despite these differences in production latencies, the analyses applied to stimulus-locked ERPs of 500 ms yielded between-group differences only in the last 100 ms. The analysis of combined stimulus- and response-locked ERPs according to individual production latencies revealed that differences across groups sustained from 400 ms after picture onset until articulation onset, which exceeded 1000 ms in the patient group.

Besides studies involving atypical populations, the comparison across participants with variable production latencies is an issue also when typical populations are investigated. In a study by Laganaro et al. (2012) using a standard picture naming task with participants issued from a homogeneous group of undergraduate students the individual mean production latencies varied from 613 to 1064 ms. The authors compared the fastest to the slowest participants and reported that ERP waveform amplitudes differed across speed-groups in the time window ranging from about 200 to 350 ms. The fast and slow initializing groups presented a similar sequence of global electrophysiological patterns from stimulus onset to articulation, except that in the 200–350 ms after picture onset the duration of stable scalp topographies varied according to production latencies. The comparison across speed groups thus indicated that a specific period of synchronized neuronal activity lasted longer in the slower participants, further shifting the following periods of stable global electrophysiological activity. Hence from 250 ms post stimulus onset ERP components are no longer aligned in fast and slow speakers. The observation of the relative duration of specific electrophysiological patterns and the consequences of shifts of components on later time periods would not be possible if only fixed time-windows were analyzed. This rationale and the methodological approach applied to the combined stimulus- and response-aligned ERPs will be further illustrated in the next section with data from a picture naming study.

## ILLUSTRATION OF ERP ANALYSES FROM STIMULUS TO SPEECH ONSET: FREQUENCY AND AGE OF ACQUISITION EFFECTS IN PICTURE NAMING

To illustrate the advantages of the procedure of ERP analysis from stimulus to response we recorded EEG during a picture naming task with pictures corresponding to high or low frequency words and to early- and late-acquired words.

### METHOD

#### Participants

The participants were 18 undergraduate students (mean age: 21.6, 3 men) who underwent this picture naming task among other tasks for course credits. They were all right-handed and French speaking with no reported neurological disease.

#### Materials and procedure

The stimuli were 60 black and white drawings and their corresponding modal names from two French databases (Alario and Ferrand, 1999; Bonin et al., 2003). Two psycholinguistic factors known to affect production latencies, namely lexical frequency and word age of acquisition (AoA), were manipulated while balancing on other pertinent psycholinguistic factors (see **Table 1**). Given the difficulty of orthogonalizing these two factors, two different experimental sets were embedded. 56 stimuli corresponded to 28 high and 28 low frequency words, matched on a set of other linguistic and pictorial factors. A second embedded set of 52 stimuli corresponded to 26 early-acquired and 26 late-acquired words, also matched on the other pertinent factors. The properties of the two sub-sets of stimuli are detailed in **Table 1**.

**Table 1:** Properties of the two sets of stimuli (high and low frequency, 56 items; early- and late-acquired, 52 items).

		Frequency set			Age of acquisition set		
		Low frequency	High frequency	*p* values	Early-acquired	Late-acquired	*p* values
**Frequency**	Movies^*^	**4.68**	**44.46**	**<0.0001**	31.02	22.14	0.42
	Text^*^	**10.11**	**66.67**	**<0.0001**	36.81	24.43	0.46
Word age of acquisition^**^		2.21	2.06	0.31	**1.73**	**2.52**	**<0.0001**
Length in phonemes^*^		4.14	4.14	1	4.19	4.09	0.73
Length in syllables^*^		1.57	1.57	1	1.69	1.47	0.19
Phono-logical neigh-borhood^*^		10.62	11.84	0.61	10.46	12.31	0.44
Onset sonority^***^		3.71	3.71	1	3.54	3.96	0.59
Name agreement^**^		96.03	94.56	0.3	95.87	94.75	0.41
Visual complexity^**^		2.57	2.71	0.59	2.45	2.87	0.11
Concept familiarity^**^		3.28	3.46	0.45	3.62	3.19	0.07

* From the database Lexique (New et al., 2004); **From Alario and Ferrand (1999) and Bonin et al. (2003) on a 5-point scale; *** on a 10-point sonority scale. Bolded values have the significant differences at *p* < 0.0001.

Participants sat approximately 70 cm in front of a PC screen in a sound-proof dimly lit room. The presentation of the trials was controlled by the E-Prime software (E-Studio). The experiment started with a familiarization phase, in which all stimuli were presented once on the screen preceded by their modal name. Then, the 60 experimental stimuli were presented twice in pseudo-random order in two blocks with a short break in between. An experimental trial started with a fixation cross presented for 500 ms, then a picture appeared on the screen and remained for 1800 ms. The participants were asked to produce the word corresponding to the picture as fast and accurate as possible. A blank screen lasting 2000 ms was displayed before the next trial. Pictures were presented in constant size (245 × 245) on a gray screen.

Word productions were digitized and production latencies (the time separating the onset of the picture and the articulation onset) were systematically checked with a speech analysis software (CheckVocal, Protopapas, 2007).

#### EEG acquisition and pre-processing

EEG was recorded continuously using the Active-Two Biosemi EEG system (Biosemi V.O.F. Amsterdam, Netherlands) from 128 channels covering the entire scalp. Signals were sampled at 512 Hz with an online band-pass filter set to 0.16–100 Hz. The custom online reference of the system is the common mode sense (CMS active electrode) – driven right leg (CMS-DRL) which drives the average potentials as close as possible to the amplifier zero (details of this setup can be found on the Biosemi website: http://www.biosemi.com). Offline, ERPs were then band-pass filtered to 0.2–30 Hz and recalculated against the average reference. ERP epochs with amplitudes exceeding ±100 μV were automatically rejected. Stimulus-aligned (i.e., forward) epochs of 500 ms and response-aligned epochs of 500 ms (i.e., backward, aligned 100 ms before production latency for each trial) were extracted. Each trial was visually inspected, and epochs contaminated by eye blinks, movements or other noise artifacts were rejected and excluded from averaging. Only trials with correct productions and valid RTs were retained and only trials with both response-aligned and its corresponding stimulus-aligned uncontaminated epochs were retained. Artifact electrodes were interpolated using 3-D splines interpolation (Perrin et al., 1987).

Stimulus-aligned and response-aligned ERPs were averaged separately per participant and subset conditions (high and low frequency words; early- and late-acquired words).

***Combining stimulus-aligned and response-aligned ERPs.*** For the topographic analyses (see below), the stimulus- and response-aligned ERPs were combined according to their corresponding production latency. To assure that there was no temporal overlap between stimulus- and response-aligned data (i.e., in cases of RTs shorter than 1100 ms), time periods of overlap were removed from response-aligned ERPs. For instance, for an average production latency of 850 ms the 500 ms stimulus-aligned ERP would be combined with response-aligned ERP data from -350 to -100 ms (see example in **Figure 1B**). In other words, the 250 ms overlapping ERP time-window would be removed from the response-aligned signal. This procedure was applied to the group averaged data per condition and on the individual ERs for each condition. As a result, the combination of stimulus- and response-aligned individual and group averaged ERPs covered the exact time interval from picture presentation to 100 ms before vocal onset in each condition. It should be noted that a similar procedure may also be applied to single stimulus- and response-aligned ERPs in case of single trial ERP analysis.

#### ERP analyses

The ERPs were first subjected to a sampling point-wise ERP waveform analysis to determine the time periods presenting local differences in ERP amplitudes between conditions. Then, a spatio-temporal segmentation was performed on the group-averaged ERPs to determine topographic differences across conditions and statistically validate them in the responses of single participants as described below.

***ERP waveform and global field power analyses.*** Electrode-wise and sampling point-wise paired t-tests served to compare the local ERP amplitudes across conditions separately on the 500 ms stimulus-aligned ERPs and on response-aligned ERPs of 300 ms (from -400 to -100 ms preceding articulation). To correct for multiple comparisons, only ERP differences present in at least five adjacent electrodes and lasting ≥20 ms were retained with a conservative alpha criterion of 0.01.

***Global topographic erp pattern analysis (spatio-temporal segmentation).*** These analyses are aimed to capture the global topographic differences across conditions. The global electric field does not change randomly either in EEG or in stimulus-elicited ERP signals; it rather remains stable over periods of tens of milliseconds before changing rapidly into a different stable configuration of the potential map (Lehmann, 1984). The aim then is to identify periods of stable global electric fields, likely corresponding to particular periods in mental information processing (Koukou and Lehmann, 1987; Lehmann et al., 1998; Changeux and Michel, 2004), and to compare them across conditions. The advantages of tracking global topographic configurations are related to their reference-independence and to their direct link to changes in the configuration of the intracranial sources (Michel et al., 2009; Michel and He, 2011). Thus, the analyses of global electrophysiological patters provide insights into how conditions differ in terms of likely underlying neurophysiological mechanisms (Murray et al., 2008; Michel and Murray, 2012), in addition to the temporal information about ERP differences. Crucially for our purpose here, the spatio-temporal analysis can be applied to ERP data of different duration. It will allow us to interpret differences across conditions observed in the voltage waveform analysis, i.e., to determine whether these differences are due to differences in the strength of the electric fields (i.e., simple modulations in amplitude), to different underlying generators (i.e., different topographies across conditions indicating changes of brain functional states) or to shifts of the ERP components across conditions. This method of analyses has been applied both to spontaneous EEG (Britz et al., 2010) and to ERPs in various cognitive domains (e.g., Pegna et al., 1997; Murray et al., 2006; Wirth et al., 2008; Knebel and Murray, 2012; Perret and Laganaro, 2012).

As a first step, we first ran a topographic analysis on each sampling point on stimulus- and backward response-aligned ERPs to identify periods of significant topographic modulation between conditions in each subset (low versus high frequency items and early- versus late-acquired words). This procedure is called TANOVA (although it is not an analysis of variance); it involves a non-parametric randomization test to the global dissimilarity between two electric fields. The global dissimilarity is a quantification of topographic differences between two electric fields independent of their strength ranging from 0 to 2 (Lehmann and Skrandies, 1984, see an example of its computation in Murray et al., 2008). The permutation of the data is accomplished by re-assigning randomly the topographic maps of single subjects to the different conditions. The global dissimilarity of these random group-averaged ERPs is compared time-point by time-point with the values of topographic dissimilarity of the actual conditions. A time-period criterion of 20 ms of consecutive significant differences was applied.

Then a global topographic ERP (map) pattern analysis called spatio-temporal segmentation was run. This procedure segments ERPs in periods of electrophysiological stability (i.e., topographic maps or ERP microstates) by compressing the variability of ERPs in a series of template maps which summarize the data and serve to determine which topographic template best explains participants’ ERP responses to each experimental condition (Murray et al., 2008; Michel and Murray, 2012). The spatio-temporal segmentation was applied to the group-averaged data (from picture onset to 100 ms before articulation) of the high and low frequency items and early- and late-acquired words. We used a modified hierarchical clustering algorithm (Pascual-Marqui et al., 1995), the agglomerative hierarchical clustering, to determine the most dominant electric field configurations at the scalp (topographic ERP maps). The selection of the optimal number of ERP maps that best explain the group-average data across conditions was based on a combination of a cross-validation and the Krzanovski–Lai criterion (see Murray et al., 2008). Statistical smoothing was applied to remove temporally isolated topographic maps with low explanatory power. This procedure is described in detail in Pascual-Marqui et al. (1995) and step by step tutorials are provided in Brunet et al. (2011) and Murray et al. (2008, see also Koenig et al., 2013). In accordance with the criteria for the local ERP waveform analyses, a given ERP topography had to be present ≥20 ms.

Then the pattern of topographic map templates observed in the group-averaged data was statistically tested by comparing each of these map templates with the moment-by-moment scalp topography (from stimulus onset to 100 ms before articulation) of individual ERPs in each condition. This procedure referred to as “fitting” allows one to establish how well a topographic template map explains single participant responses in each condition. Each data sampling point in each condition was labeled according to the template map with which it best correlated spatially, yielding a measure of map presence in milliseconds and of global explained variance. These measures are then used to statistically test topographic differences across conditions.

### RESULTS

#### Behavioral results

Mean production accuracy was 95.8% and mean production latency (reaction time, RT) was 858 ms (range of mean RT per participant per condition: 703–1096).

Early-acquired words were initialized 63 ms faster than late-acquired words [*t*(17) = -9.8, *p* < 0.0001], whereas RTs did not vary for high versus low frequency words (see **Table 2**).

**Table 2:** Mean production latencies in ms and mean error rate in brackets for each set of stimuli.

Frequency manipulation set		AoA manipulation set	
Low frequency	High frequency	Early-acquired	Late-acquired
857 (3%)	859 (4%)	826 (3%)	890 (5%)

#### ERP results

**FIGURE 2:**
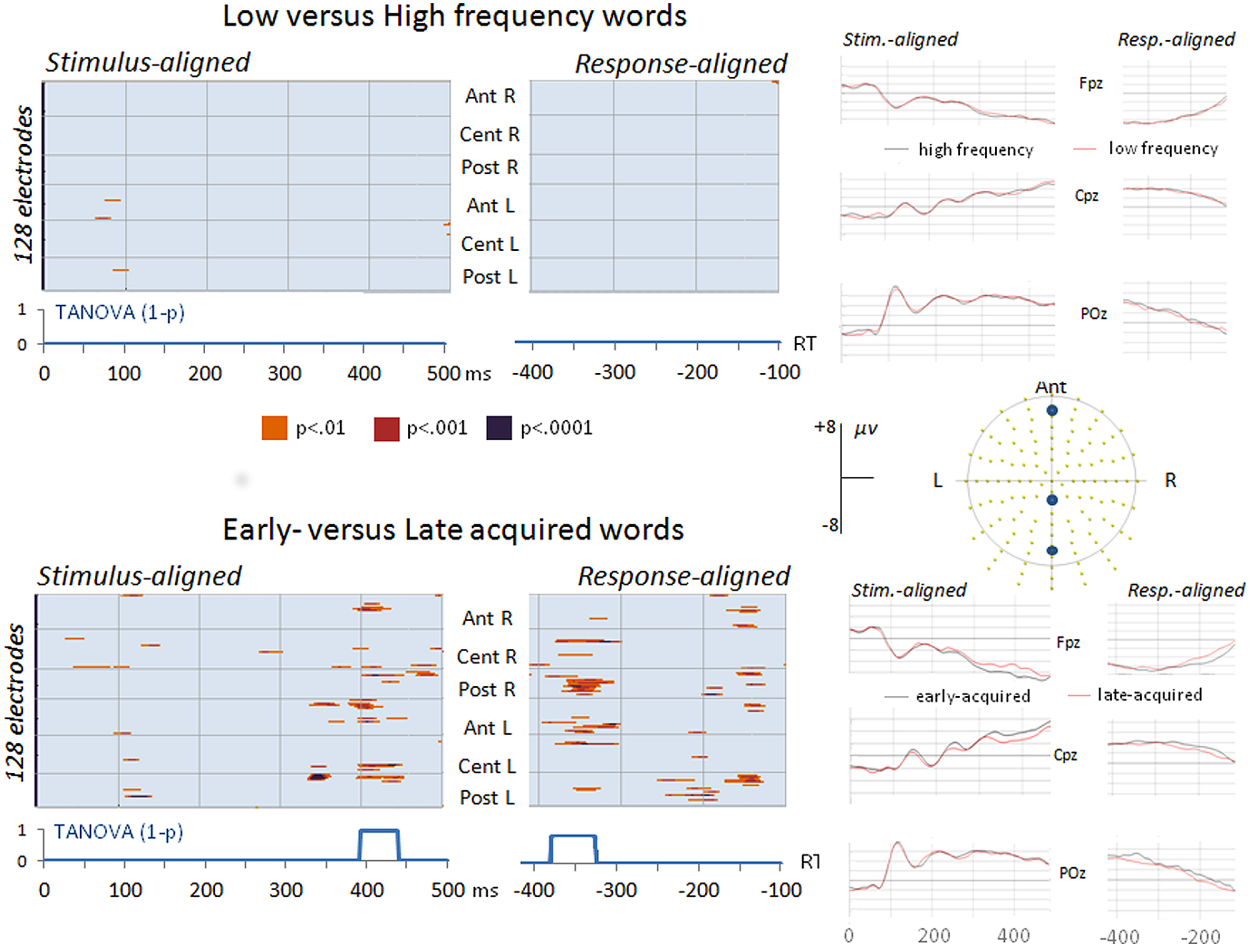
**Significant differences (*p* values) on ERP waveform amplitudes on each electrode (*Y* axes) and time point (*X* axes) on the stimulus-aligned and response-aligned ERPs between low and high frequency words (top) and between early- and late-acquired words (bottom) with results of the topographic TANOVA analyses (1 *– p* values) and display of three averaged stimulus-aligned and response-aligned ERP waveforms (Fpz, Cpz, and POz) in each condition**.

The comparison between high and low frequency items revealed no differences on amplitudes or on global dissimilarity neither on the response-aligned nor on the stimulus-aligned ERPs (see top of **Figure 2**). In the analysis of early- and late-acquired words diverging amplitudes appeared on more than 20 electrodes from about 400–450 ms in the stimulus-aligned ERPs and in two time windows in the response-aligned ERPs: between 380 and 320 ms before articulation onset and around 150 ms before articulation (**Figure 2**). The global dissimilarity (TANOVA) analysis also revealed significant differences between early- and late-acquired words in the same time-windows.

**FIGURE 3:**
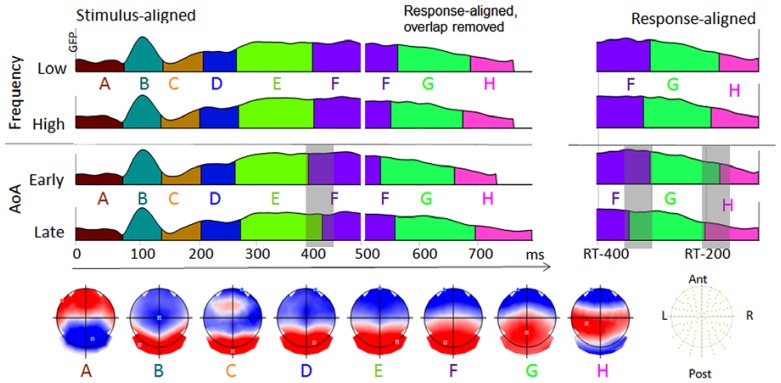
**The temporal distribution of the topographic maps revealed by the spatio-temporal segmentation analysis is displayed with different colors under the GFP of the stimulus-aligned and response-aligned grand average ERP of each condition.** The corresponding template maps are displayed with positive values in red and negative values in blue and with display of the maxima and minima of scalp field potentials.

The spatio-temporal segmentation applied to the four grand averaged ERPs from picture onset to 100 ms before voice onset revealed eight different topographic patterns accounting for the 97% of the variance. This specific cluster corresponded to a minimum peak in the cross validation criterion and a maximum in the modified Krzanowski–Lai. The distribution of the periods of stable topographic activity are displayed in **Figure 3** for the stimulus-aligned ERPs, the response-aligned ERPs and their combination according to the mean production latencies in each condition (colors under the global fit power – GFP-code periods of stable topographies).

In the first period corresponding to different amplitudes and TANOVA in **Figure 2** (around 400–450 ms) different topographic maps appeared between early- and late-acquired words. These differences seem to be due to a shift of a period of global electrophysiological stability (map labeled “E” lasting shorter in early- than in late-acquired words). To verify whether this shift is consistent across participants, maps E and F were fitted in the time-period between 300 and 500 ms in each individual data per condition. The mean duration of map “E” across participants in this fitting period was 94 ms for early-acquired words with 41% of the global explained variance (GEV) and 121 ms with 51% of explained variance for late-acquired words (Wilcoxon *z* = -2.22, *p* < 0.05 on duration and *z* = -2.24, *p* < 0.05 on GEV). If we refer to the *fixed* time-windows of both the stimulus-aligned or the response-aligned ERPs (from -400 to -100 ms preceding articulation, on the right in **Figure 3**) the following period of stable electrophysiological pattern (map “F”) seems to last longer in the early-acquired than in the late-acquired condition (109 versus 80 ms in the fitting in the response-aligned ERPs, Wilcoxon *z* = -2.3, *p* = 0.02). However, the situation looks differently when considering the combined stimulus- and response-aligned ERPs covering the exact production latencies in each condition (with overlapping signal removed from the response-aligned ERPs). The stable topographic pattern “F” is shifted in the late-acquired condition because of longer duration of the previous pattern “E,” but it has absolute similar duration across conditions. Indeed, the fitting of maps E, F, G, H from 300 ms after picture onset to 100 ms before articulation for each participant and condition revealed no significant difference in the overall duration of map “F” across early- and late-acquired words (respectively 150 and 143 ms, Wilcoxon *z* < 1). Finally, the last period of topographic stability (labeled “H” in **Figure 3**) had longer duration for late-acquired words than for early-acquired words (respectively 102 and 72 ms, Wilcoxon *z* = -2.34, *p* < 0.02), but had similar GEV (11 versus 10%, *z* < 1).

## DISCUSSION

Here we presented the problems related with the analysis of fixed ERP time-windows in speech production studies with variable initializing latencies. We then proposed a method combining stimulus-locked and response-locked ERPs in order to cover the whole speech planning process according to production speed and we illustrated this approach coupled with topographic analysis by means of a picture naming study.

In the illustration, word age of acquisition (AoA) modulated local waveform amplitudes and global topographies after 400 ms, but only the topographic analyses run on the ERPs covering the actual production latencies in each condition led to a coherent interpretation of the underlying neurophysiological difference between early- and late-acquired words.

We will only briefly discuss the observed effects of the manipulated factors in relationship with word production models to keep the focus on the purpose of the paper, namely the illustration of the approach.

### EFFECTS OF THE MANIPULATED FACTORS (LEXICAL FREQUENCY AND AGE OF ACQUISITION)

Among the two linguistic factors manipulated in the two subsets of stimuli, only AoA affected production latencies and ERPs. Despite the careful manipulation of both written and spoken frequency values, no lexical frequency effects were observed. At first glance the present results seem in contradiction with two picture naming studies by Strijkers et al. (2010, 2011), who reported lexical frequency effects on both production latencies and waveform amplitudes using a similar picture naming factorial design. However, the absence of lexical frequency effects on production latencies in picture naming studies has been repeatedly reported, in particular when stimuli were matched on a large set of other pertinent factors including word age of acquisition (Morrison et al., 1992; Snodgrass and Yuditsky, 1996; Barry et al., 2001; Bonin et al., 2003). A careful comparison between the present study and the two studies by Strijkers et al. (2010), 2011 reveals a very different operationalization of other psycholinguistic factors. In the latter studies, high and low frequency stimuli were matched on a restricted set of pertinent factors (visual complexity of the pictures and word length in Strijkers et al., 2010; length and name agreement in Strijkers et al., 2011) whereas other variables such as age of acquisition, phonological neighborhood, and concept familiarity were not considered. Hence, the absence of lexical frequency effects both on production latencies and on ERPs in the present study possibly relies on the accurate match on a large set of other linguistic factors as reported in previous chronometric studies.

Word age of acquisition effects on production latencies have been more consistently reported even when stimuli were matched on a large set of other possible confound variables in factorial designs (Barry et al., 2001) as well as with multiple regression approaches (Snodgrass and Yuditsky, 1996; Bonin et al., 2003; Alario et al., 2004). In the present study late-acquired words were produced 64 ms slower than early-acquired words. In addition, AoA modulated ERPs on both amplitudes and periods of stable topographic patterns after 400 ms following picture onset. This observation replicates with different stimuli and participants previous results on AoA modulations on ERPs in picture naming tasks (Laganaro and Perret, 2011; Laganaro et al., 2012; Perret et al., 2014). In all these studies AoA effects have been reported on ERPs from around 400 ms after picture presentation. This time-window has been associated with later word planning processes, likely lexical-phonological encoding (Indefrey and Levelt, 2004; Indefrey, 2011), which converges with the locus of AoA effect determined in behavioral studies (Morrison et al., 1992; Chalard and Bonin, 2006; Kittredge et al., 2008). The interpretation of the observed ERP modulation in terms of neurophysiological processes is contingent to the methodological approach and we will discuss it closely in the next section.

### ANALYSES ON SEPARATE VERSUS COMBINED STIMULUS- AND RESPONSE-ALIGNED ERPs

The different waveform amplitudes observed after 400 ms between early- and late-acquired words do not inform us about the nature of the underlying difference. There are indeed several possible interpretations of these differences. Planning early- and late-acquired words may recruit different neuronal networks, suggesting that they are encoded via different brain processes after 400 ms; alternatively, early- and late-acquired words may recruit the same neuronal circuits but with different strengths; finally, the difference between early- and late-acquired words may stem from a shift of identical underlying brain mechanisms, indicating that a given process takes more time in one condition relative to the other. The results of the spatio-temporal segmentation favor the third interpretation, as the same sequences of stable topographic configurations of the potential fields on the scalp appeared in all conditions but with different distributions (different durations).

Crucially for the purpose here, the separate analysis of stimulus- and response-aligned ERPs and the analysis of the combined ERP signal covering the exact production latency led to two very different interpretations of the results. In the separate analysis of fixed stimulus- and response-locked ERPs a stable topographic configuration (map “F” in **Figure 3**) had longer duration in the early-acquired word data than in the late-acquired words, whereas maps “E” and “H” had longer duration for late-acquired words than for early-acquired words. Based on these observations, one may conclude that from about 400 ms post stimulus onset two specific periods of stable electrophysiological activity last longer for late-acquired words, whereas the opposite is observed for the brain process corresponding to map “F,” which lasts about 50 ms longer for early-acquired words. This latter result is counter-intuitive as longer lasting neurophysiological processes when planning early-acquired words would hardly be interpretable in the light of faster production latencies for these words.

This situation is disambiguated in the analysis of combined stimulus- and response-locked ERPs according to the actual production latencies, which enabled the observation that the period of stable electrophysiological activity corresponding to map F was shifted in late-acquired words by the previous longer lasting period of topographic stability (map E in **Figure 3**), but that it had similar overall duration across conditions. The counter-intuitive results observed in the analysis of fixed ERP time-windows were due to the fact that they captured different portions of the planning process across the faster and slower conditions.

This illustration clearly shows that coherent results on ERP modulations in later time-windows only appeared when combining stimulus and response-aligned ERPs in order to cover the actual RT. It also allows ERP analyses to capture the consequences of earlier modulations on later ERP components: here the shift of later processes due to a longer lasting stable electrophysiological pattern in the stimulus-aligned data. In addition, although the first shift across conditions may be identified by visual inspection of some waveforms (see for instance shift of a P3-like component on Cpz in **Figure 2**), the following shifts could only be apprehended with the global topographic analysis.

## CONCLUDING REMARKS

Here we showed that a coherent interpretation of the results only arose when combining stimulus- and response-aligned ERPs in order to cover the actual word planning time-window in each participant and condition. This combination enabled us to disentangle the possible interpretations of the neurophysiological processes underlying differences across conditions observed on waveform and on topographies in the fixed ERP periods. This approach is very useful in language production studies when comparing conditions or populations presenting different production latencies. Whereas ERPs clearly present sequences of stable periods of global electrophysiological activity enabling the comparison across conditions or groups in terms of global topographic patterns, further research is needed to improve our understanding of the relationship between the observed periods of topographic stability and the mental operations involved in speech planning.

## Conflict of Interest Statement

The author declares that the research was conducted in the absence of any commercial or financial relationships that could be construed as a potential conflict of interest.
